# Supplementary material for: A barbed end interference mechanism reveals how capping protein promotes nucleation in branched actin networks
Source: Nat Commun. 2021 Sep 9;12:5329. doi: 10.1038/s41467-021-25682-5 (PMC8429771; doi:10.1038/s41467-021-25682-5)
Supplement: Supplementary file 1 — Supplementary Information [file 41467_2021_25682_MOESM1_ESM.pdf]

<u>Supplementary Information</u> for

“A barbed end interference mechanism reveals how capping protein promotes nucleation in branched actin networks”

Supplementary Figures 1-9

Supplementary Tables 1-4

Supplementary Figures

Supplementary Figure 1

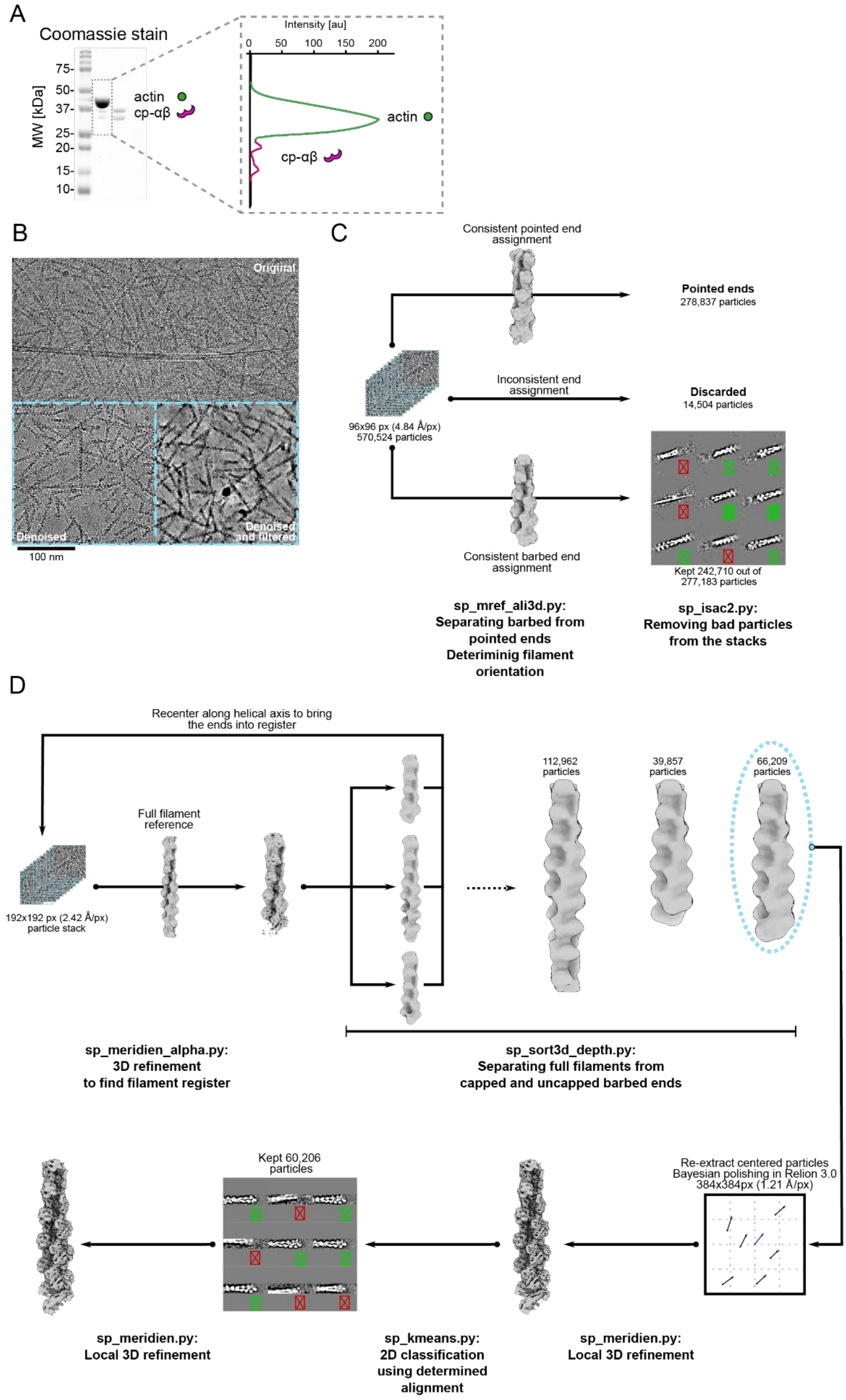

Supplementary Figure 1: Structure determination of CP-bound filament barbed ends. (A) Protein gel from size-exclusion chromatography. Left: Original Coomassie-stain with visible bands representing actin and α, β-capping protein (see Figure 1 B). Right: Intensity plot of representative protein bands for actin (green) and capping protein (magenta). The experiment was repeated at least 5 times with similar results. (B) – (D) Image processing strategy used to obtain the Cryo-EM map of capped filaments. (B) Representative micrograph imaged at ~ -1.5 µm defocus. Similar results were obtained in at least five independent experimental replicates. The contrast of the images was enhanced with the neural-network denoiser janni, and subsequently with a ctf-correction filter. This was necessary to train a robust crYOLO model for autopicking. (C) Pointed and barbed ends were separated using 3D multi-reference alignment. Only those particles that were assigned consistently in two independent runs were labeled as either pointed or barbed ends. (D) 3D-refinement strategy. 3D-refinement runs were started with a full filament as reference. Initially, the filament ends were located in different monomers along the filament. Using focused 3D classification, we separated them, brought them into register, and used them for a new round of 3D refinement. After the final classification, a single population of capped ends could be identified. Those were subjected to Bayesian polishing[1], and further refined. A final step of k-means classification using the previously determined euler angles served to remove the last remaining back picks. This was followed by a final local refinement to produce the final map.

Supplementary Figure 2

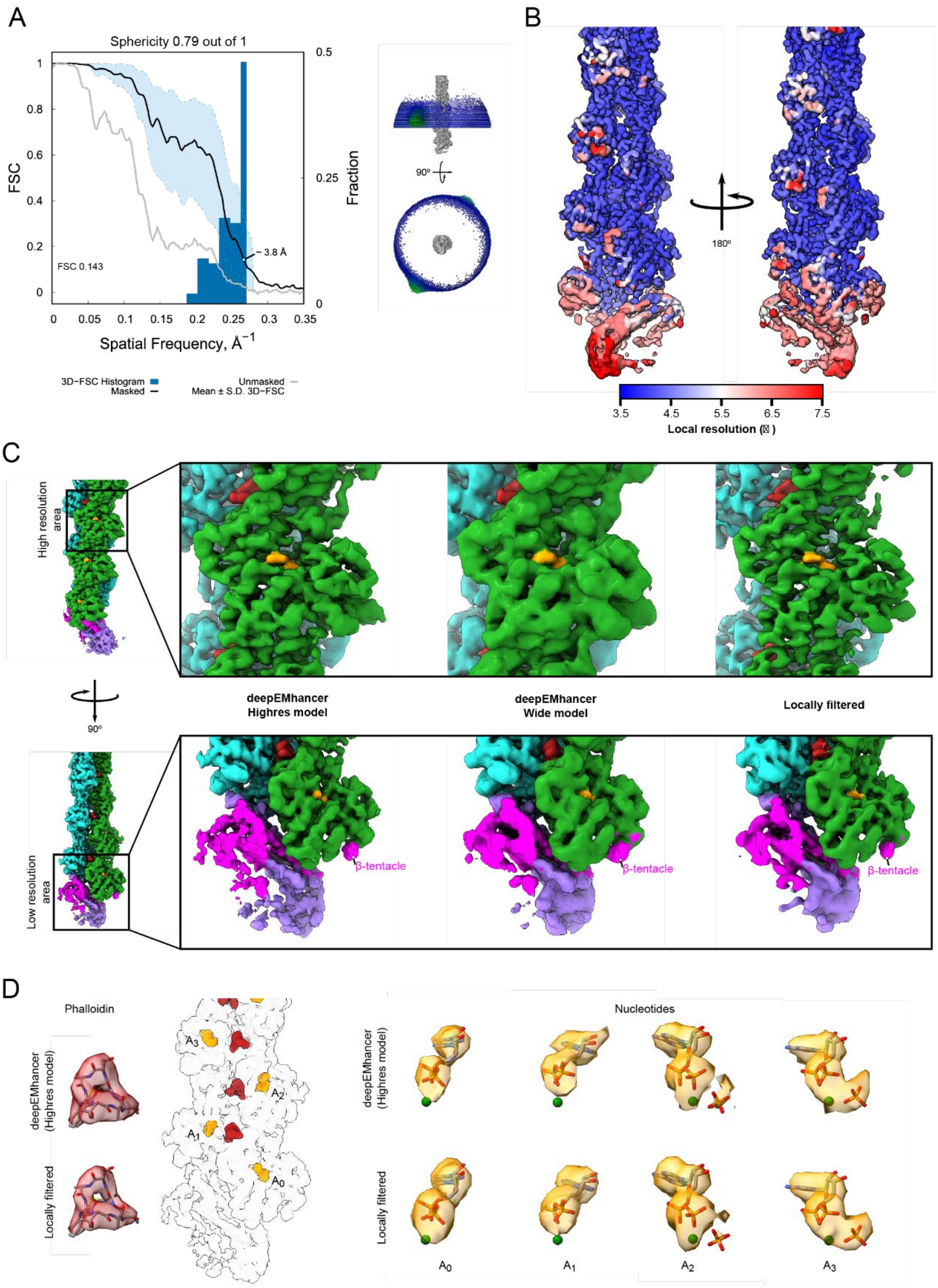

Supplementary Figure 2: Resolution estimation for the Cryo-EM map. (A) Resolution estimation using Fourier shell correlations. The solid black and gray lines show the overall FSC curves for the map with or without applying a soft mask. The dashed light blue lines and the region enclosed by them represent the average ± one standard deviation of the directional masked FSC curves calculated by 3D-FSC [2]. A moderate preferential orientation is visible in

these curves as well as in the distribution of Euler angles shown on the right of the plot. (B) Local resolution estimated using the false discovery rate procedure implemented in SPOC[3]. The color of the map, post-processed by deepEMhancer, goes from low resolution in red to highest resolution in blue. (C) Appearance of the map in regions of high or low local resolution. For each region, maps post-processed with the highres or wide models of deepEMhancer or locally filtered with SPOC are presented. Colors are is in Figure 2. (D) Small molecule density found in the Cryo-EM map. The map in the center highlights the position of the small molecules found along the filament. (left) Representative phalloidin density. (right) Density at the nucleotide-binding site of the first 4 actin monomers. Clear density for ADP can be seen in all of them. The two terminal protomers lack additional density for P_i, which is present in all remaining active sites. The panels include the density coming from maps post-processed with deepEMhancer or locally filtered.

Supplementary Figure 3

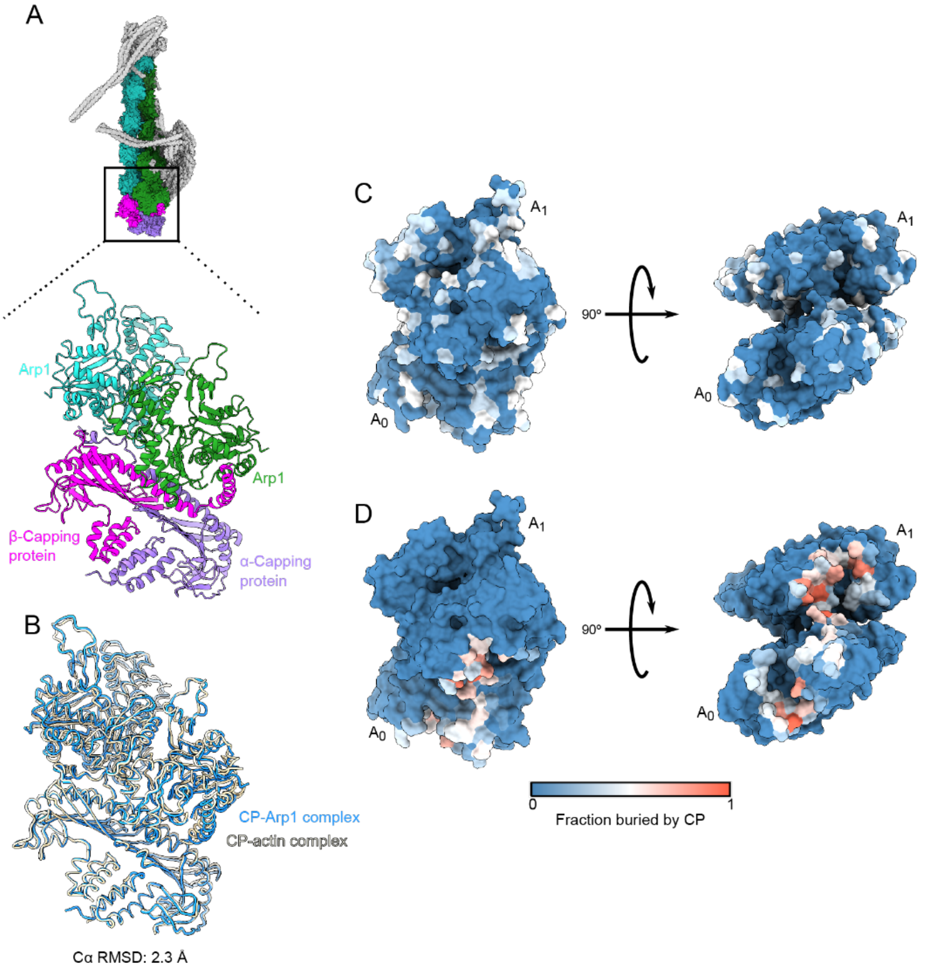

Supplementary Figure 3. **Comparison between dynactin and capped actin filaments.** (A) Structure of the dynactin complex[4] (PDBID 5ADX [https://www.rcsb.org/structure/5adx]). For clarity, we colored only capping protein and the Arp1 filament, leaving the rest of the complex in silver. Colors are as in Fig 2. The zoomed region shows a ribbon representation of the last two barbed end Arp1 protomers and capping protein. (B) Superposition of the two terminal filament subunits of dynactin's Arp1 and actin, together with their corresponding capping protein. (C) Similarity between *B. taurus*' β-actin and Arp1. Identical residues are colored in blue while increasing dissimilarity is depicted a lighter color. Similarity was calculated with Multiseq within VMD (entropy similar) (D) Per-residue fraction of actin's area buried by CP.

Supplementary Figure 4

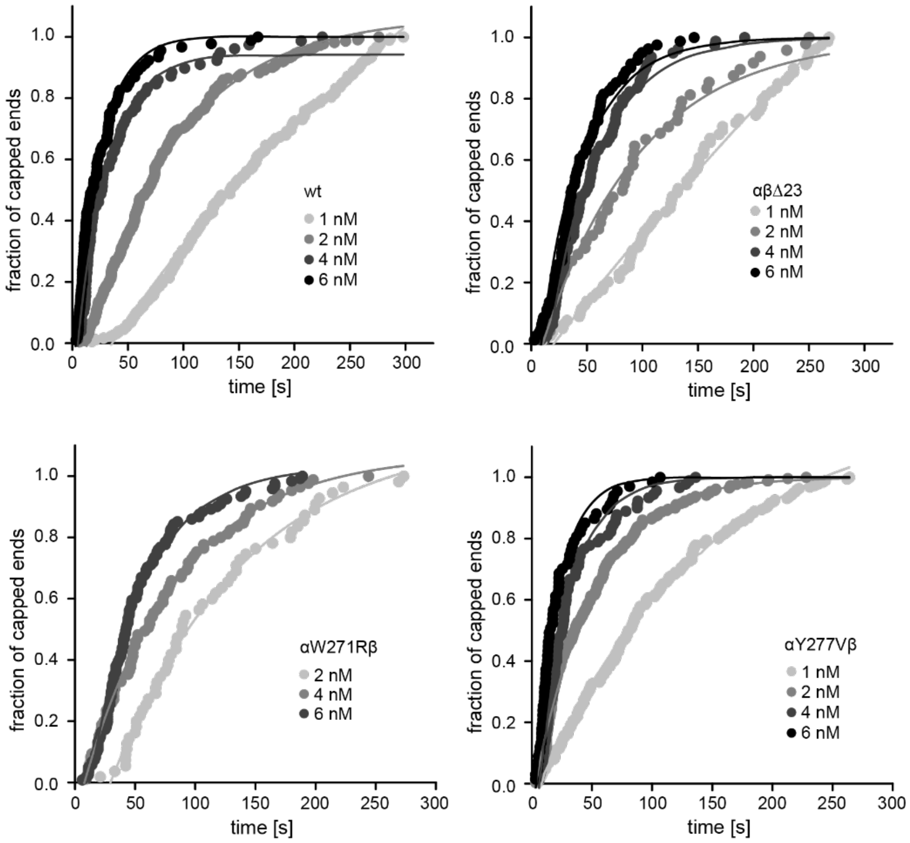

Supplementary Figure 4: Determination of the barbed end association rate constants for capping protein wt and mutants. Rate of actin filament capping in the presence of 2 µM Mg-ATP-actin (10 nM Alexa488-lifeact), 2 µM profilin1, 1–6 nM capping protein (wt or mutant as indicated, N ≥ 80 filaments tracked). Examples of individual time course are shown including fits to a mono-exponential growth function (see Methods). The observed reaction rates (k_{obs}) (plotted in Figure 3G) were derived from the mean of N=3 independent experiments for each CP concentration. Source data are provided as a Source Data file.

Supplementary Figure 5

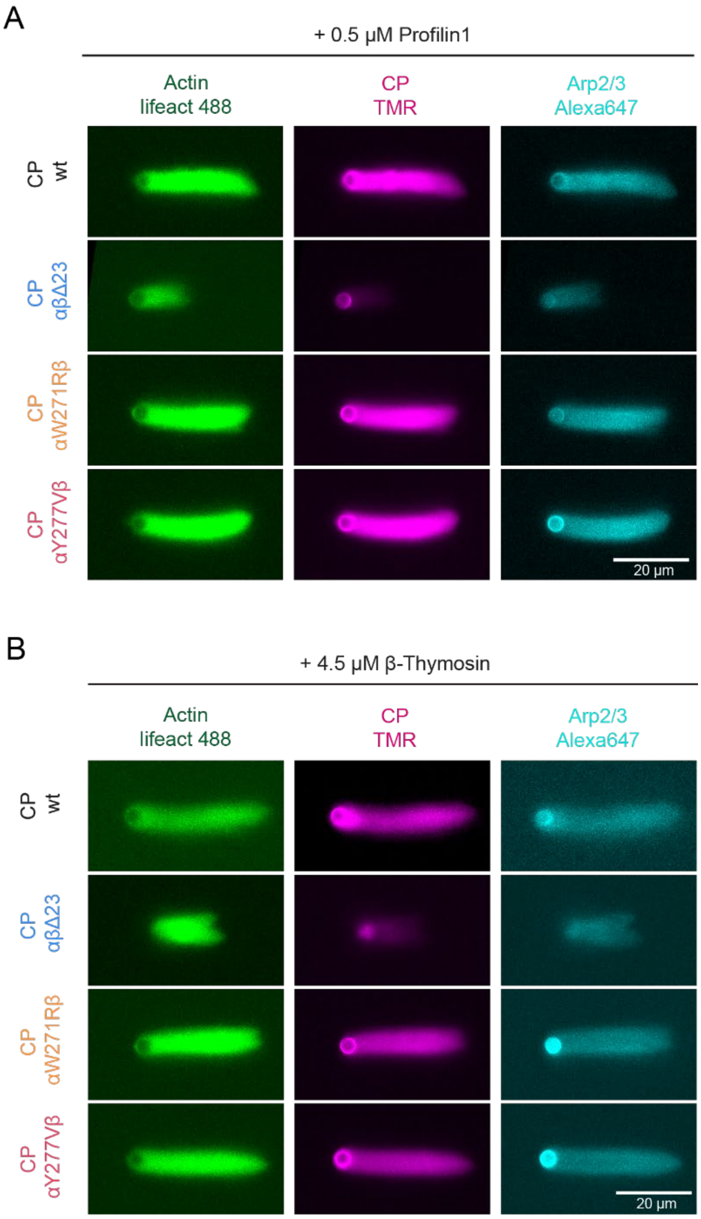

Supplementary Figure 5: Reduction of free actin monomer levels by addition of excess profilin or thymosin-β4 does not alter branched network assembly (A) Epifluorescence images of dendritic actin networks grown from WAVE1ΔN-coated microspheres using 5 µM profilin–actin complexes plus 0.5 µM profilin (see Methods), 100 nM CP (either wt or mutants as indicated, 20 % TMR-labeled), 100 nM Arp2/3 (20 %-Alexa647 labeled). Reactions were kinetically arrested after 4 min using phalloidin (15 µM), latrunculin-B (15 µM) and Alexa488-lifeact (15 nM) to visualize filamentous actin. The experiment was repeated three times with similar results. (B) Same as A but with 4.5 µM thymosin-β4 added to 5 µM profilin–actin. The experiment was repeated three times with similar results.

Supplementary Figure 6

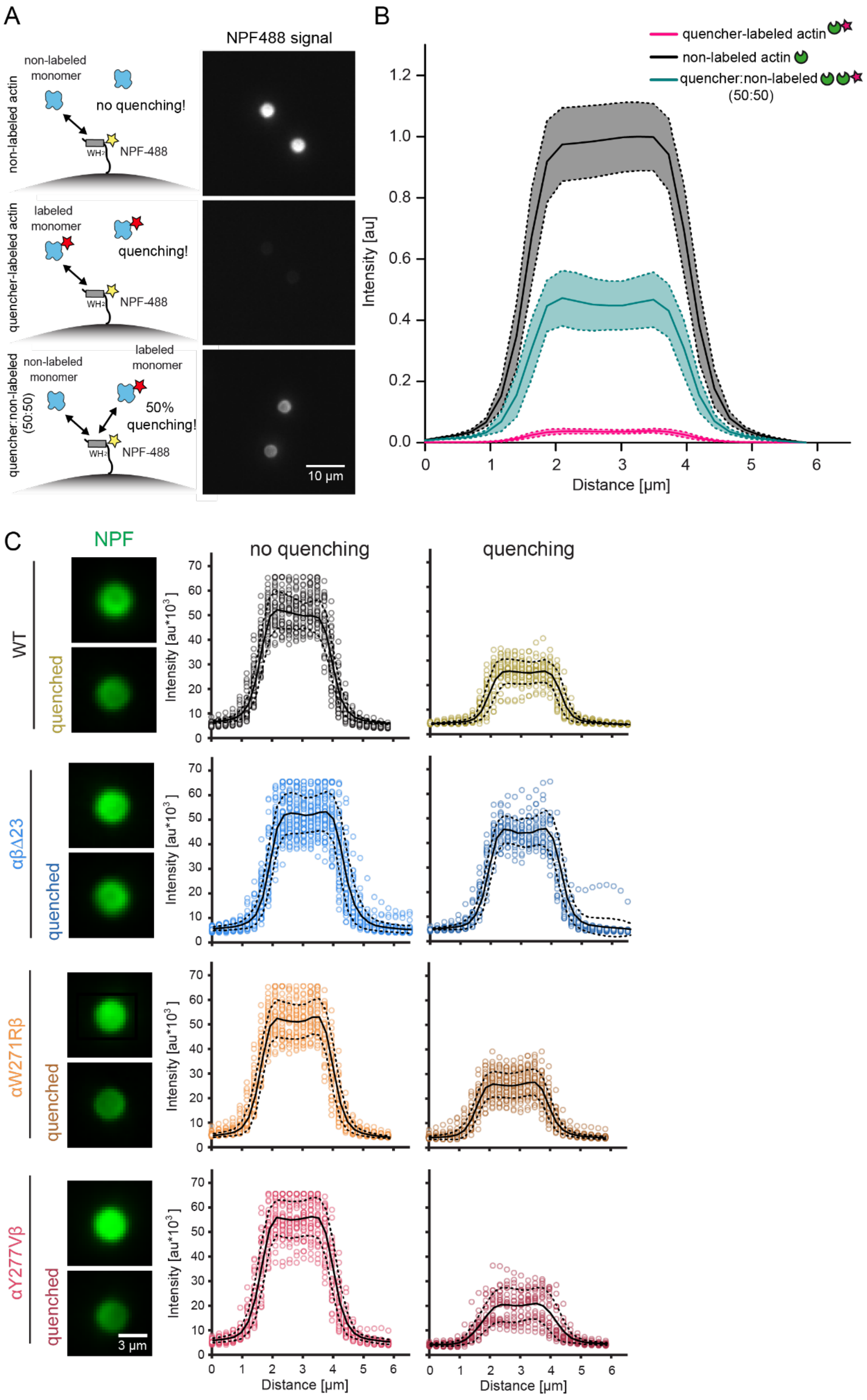

Supplementary Figure 6: Control experiments for NPF-fluorescence quenching by quencher-labeled actin monomers in bead motility assays. (A) Left: Scheme of experimental setup. Biotinylated, Alexa-488-labeled NPF molecules were bound to the surface of microspheres by a streptavidin linker. The NPF-coated microspheres were incubated with unlabeled (top), quencher-labeled (middle) or a mix of labeled and unlabeled (50:50, bottom) actin monomers, under non-polymerizing conditions, as indicated. Right: Representative images of the NPF-Alexa488 fluorescence signal intensity. (B) Average NPF-fluorescence signal intensities (with error = SD) from NPF-coated microspheres incubated with non-labeled and quencher-labeled actin monomers as indicated in (A). (n=45 beads per condition). Source data are provided as a Source Data file. (C) Left: Representative images from NPF-Alexa488-coated microspheres (see Figure5B) acquired in wide field epifluorescence 3 min after arrest before and after quenching with quencher-labeled actin monomers. Networks were grown in presence of wt or mutant capping proteins, as indicated. Right: single point intensities (dots) and average intensities (lines, with error = SD/dotted lines) of the NPF-fluorescent signal intensity on the microsphere surface in presence (quenching) and absence (no quenching) of quencher-labeled actin monomers (n=25 beads per CP-type and condition). Source data are provided as a Source Data file.

Supplementary Figure 7

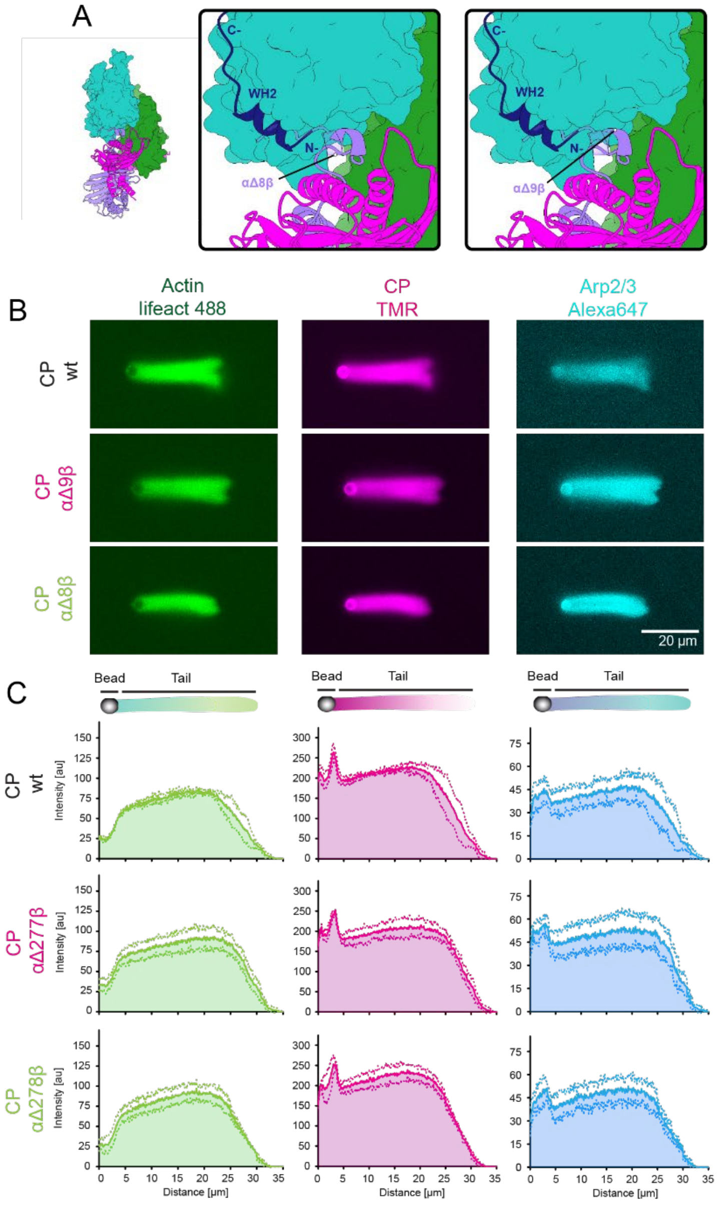

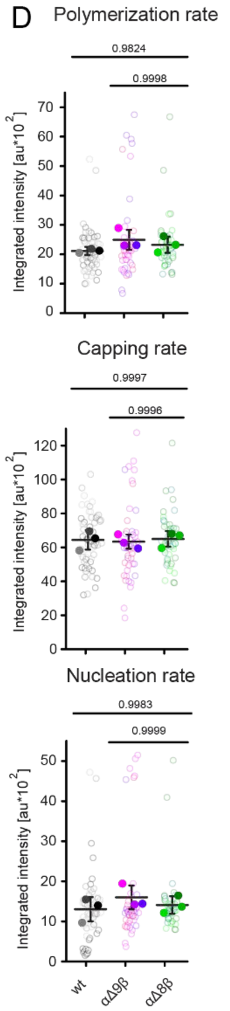

E

Integrated intensity [au*10^2]		wt	αΔ9β	αΔ8β
	Actin-488	21 ± 1	25 ± 3	23 ± 3
	CP-TMR	64 ± 6	63 ± 4	65 ± 5
	Arp2/3-647	13 ± 3	16 ± 3	14 ± 2

Supplementary Figure 7: The extreme terminus of the CP α tentacle is dispensable for reconstituted branched actin network assembly. A) Overview of the location of the CP α tentacle deletions at the penultimate actin subunit in relation to the putative NPF WH2 binding site. For guidance, N- and C-terminal ends of the WH2 fragment are labelled. Colors are as in Fig 2 (B) Epifluorescence images of dendritic actin networks grown from WAVE1ΔN-coated microspheres using 5 μM profilin–actin complexes (see Methods), 100 nM CP (either wt or mutants as indicated, 20 % TMR-labeled), 100 nM Arp2/3 (20 %-Alexa647 labeled). Reactions were kinetically arrested after 4 min using phalloidin (15 μM), latrunculin-B (15 μM) and Alexa488-lifeact (15 nM) to visualize filamentous actin. The experiment was repeated three times with similar results. (C) Quantification of the averaged intensity profiles for the indicated dendritic network components for n=25 actin networks from N=3 independent experiments, error indicator = SD of the mean. Source data are provided as a Source Data file. (D) Plots of the polymerization, capping and nucleation rates of dendritic actin networks grown in presence of either wt or mutant CP (see Methods). Quantifications were done for n=25 actin networks from N=3 independent experiments, error indicator = SD of the mean. P-values (D) were derived from one-way ANOVA Tukey tests. Source data are provided as a Source Data file. (E) Mean integrated intensities of actin, capping protein and Arp2/3 complex fluorescence signals for dendritic actin networks grown in the presence of wt or mutant capping protein as indicated. Quantifications were done for n=25 actin networks from N=3 independent experiments, error indicator = SD of the mean.

Supplementary Figure 8

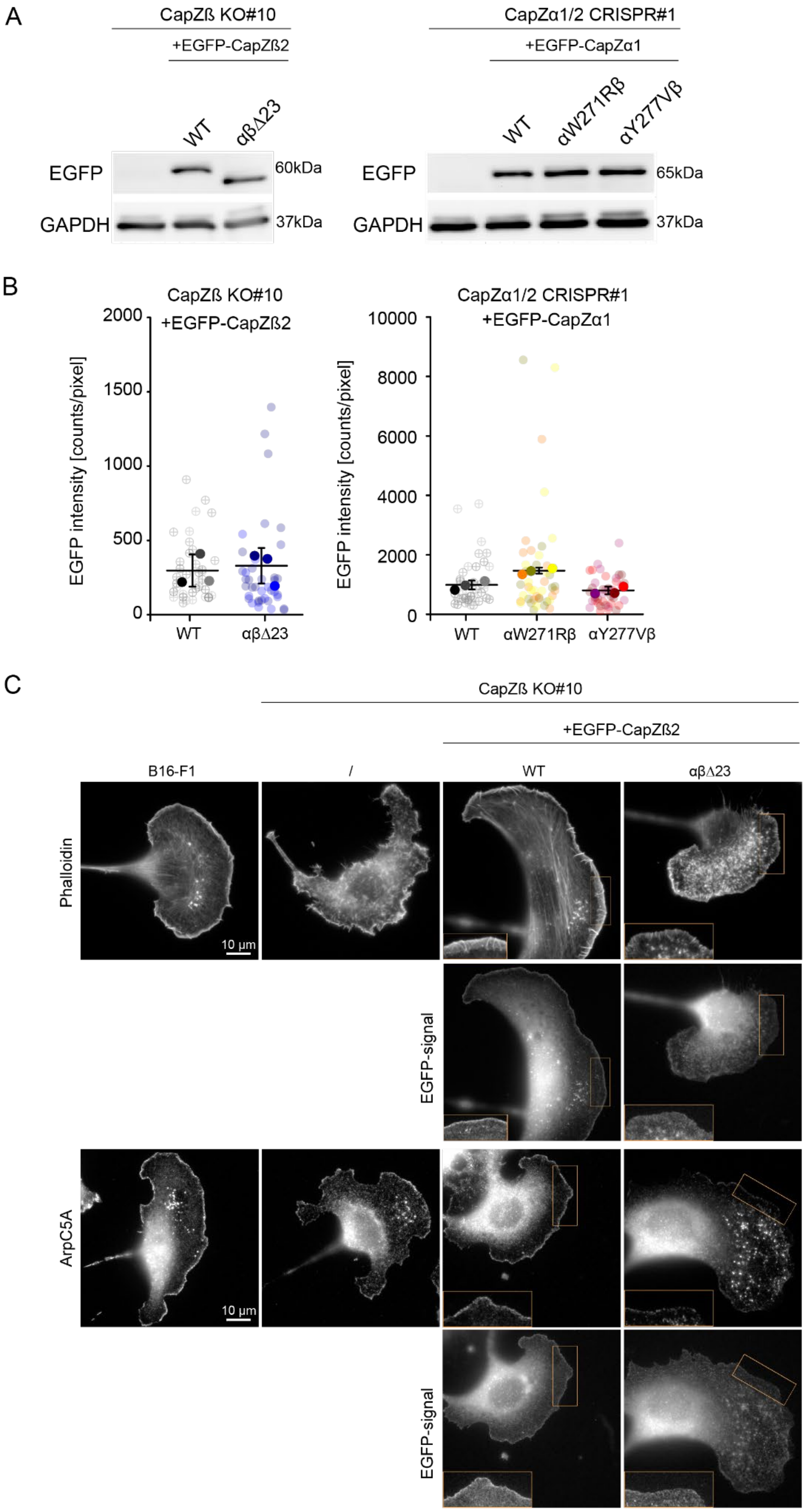

Supplementary Figure 8: Expression levels of EGFP-tagged CP rescue constructs, and actin filament and Arp2/3 complex densities at the leading edge under select CP rescue conditions. (A) Lysates of CapZβ KO (clone #10, left) and CapZα1/2 CRISPR/Cas9-treated cells (clone #1, right) untransfected or transfected with constructs as indicated were subjected to Western blotting using anti EGFP antibody. The experiment was performed once. (B) Single cell EGFP fluorescence intensity measurements of ectopically expressed CapZβ2 (left) and CapZα1 (right) EGFP-fusion constructs, as obtained from live cell imaging (see Methods). Quantifications were done for n=39 cells from N=3 independent experiments, error indicator = SD of the mean. Source data are provided as a Source Data file. (C) Representative cell morphologies of B16-F1 and CapZβ KO cells (clone #10) untransfected or transfected with indicated, EGFP-tagged CapZβ2 constructs. Samples were stained for the actin cytoskeleton with phalloidin (top) or the Arp2/3 complex using anti-ArpC5A antibody (bottom). In case of rescue experiments (right), boxed regions (orange) highlight magnified insets shown at the bottom left of each panel to reveal localisation patterns of F-actin and Arp2/3 complex as compared to respective, EGFP-tagged rescue construct. Representative data is shown from two independent experiments. The experiment was repeated two times with similar results. Scale bars = 10 µm.

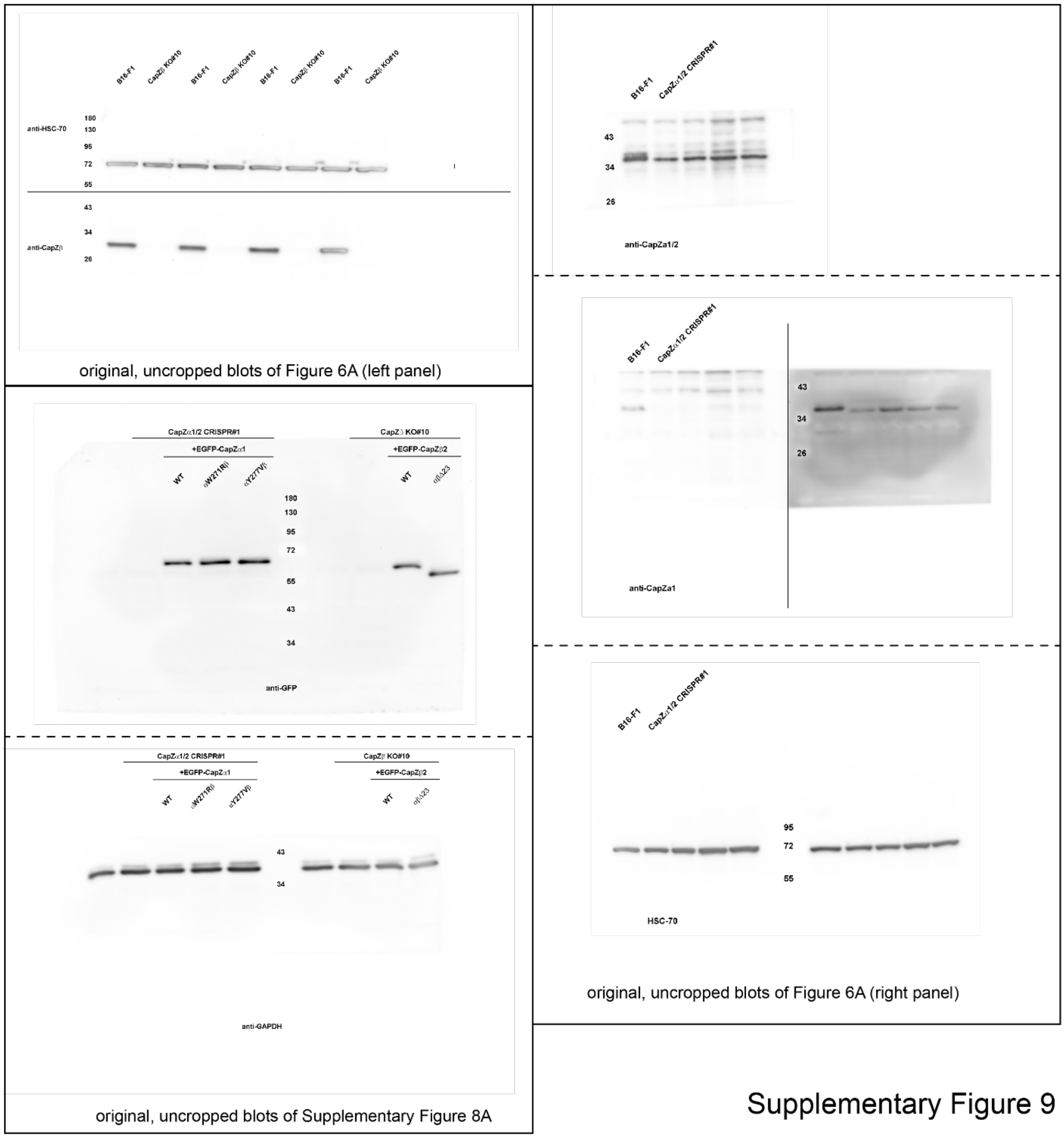

Supplementary Figure 9: Original blots. Original, uncropped images of blots utilized in Figure 6 and Supplementary Figure 8 as indicated.

Supplementary Table 1

	(EMDB- 13343)<br>(PDB 7PDZ)
**Data collection and processing**	
Magnification	120,000x
Voltage (kV)	200
Electron exposure (e–/Å^2)	60
Defocus range (μm)	-0.73 - -4.0
Pixel size (Å)	1.21
Symmetry imposed	C1
Initial particle images (no.)	570,724
Final particle images (no.)	60,206
Map resolution (Å)	3.8
FSC threshold	0.143
Map resolution range (Å)	3.5 - 7.5
**Refinement**	
Initial model used (PDB code)	5ADX
Model resolution (Å)	4.0
FSC threshold	0.5
Model resolution range (Å)	3.5 - 7.5
Map sharpening *B* factor (Å^2)	-40
Model composition	
Non-hydrogen atoms	22,186
Protein residues	21,998
Ligands	188
B factors (Å^2)	
Protein	64-26 -
Ligand	
R.m.s. deviations	
Bond lengths (Å)	0.02
Bond angles (°)	1.928
Validation	
MolProbity score	1.63
Clashscore	4.64
Poor rotamers (%)	0.21
Ramachandran plot	
Favored (%)	94.04
Allowed (%)	5.92
Disallowed (%)	0.004

Supplementary Table 1. Statistics of the Cryo-EM model of capped actin filaments.

Supplementary Table 2

		wt	αβΔ23	αW271Rβ	αY277Vβ
Integrated intensity [au*10⁴]	Actin-488	67 ± 3	30 ± 7	65 ± 7	70 ± 10
	CP-TMR	82 ± 5	4 ± 2	83 ± 4	85 ± 9
	Arp2/3-647	37 ± 2	12 ± 0.2	38 ± 2	41 ± 4
network growth rate [µm/min]		4.9 ± 0.2	2.0 ± 0.1	4.9 ± 0.4	5.1 ± 0.2

Supplementary Table 2. Mean integrated intensities of fluorescence signals derived from actin, capping protein and Arp2/3 complex in dendritic actin networks grown in the presence of wt or indicated mutant capping protein. Quantifications were done for n=25 actin networks from N=3 independent experiments, error indicator = SD of the mean. Network growth rates (µm/min) for each condition are shown at the bottom.

Supplementary Table 3

**Primer name**	**Primer sequence (5'→3')**
CPalpha fwd	AATTAAGATCTGGTGGAGGTGGTTCTATGGCCGACTTTGAGGATCG
CPalpha rev	AATTAGGTACCTTATTAAGCATTCTGCATTTCTTTGC
CPalpha d8 rev	AATTAGGTACCTTATTACTTGTAACTGAGTATCTTGTTCC
CPalpha d9 rev	AATTAGGTACCTTATTAAGTAACTGAGTATCTTGTTCCAG
CapZb_delta23_fwd	GTCTGTGCAGACGTTTTGAGACAAATCAAAGCAAG
CapZb_delta23_rev	CTTGCTTTGATTTGTCTCAAAACGTCTGCACAGAC
Cas9_ CapZa1_fwd	CACCGAGTTTAATGAAGTATTCAA
Cas9_ CapZa1_rev	AAACTTGAATACTTCATTAAACTC
Cas9_ CapZa2_fwd	CACCGCAGAAGGAAGATGGCGGATC
Cas9_ CapZa2_rev	AAACGATCCGCCATCTTCCTTCTGC
Cas9_ CapZb_fwd	CACCGCCTCAGCGATCTGATCGACC
Cas9_ CapZb_rev	AAACGGTCGATCAGATCGCTGAGGC
CapZa1_PCR_fwd	CTGGGCACTGCATGTACAAA
CapZa1_PCR_rev	CGACCAGTGAAGAGTACGCA
CapZa2_PCR_fwd	GGAAGACCGCTCGGAAAGG
CapZa2_PCR_rev	CACCGAGGCGTGAATTCCT
CapZb_PCR_fwd	CACCTTGGTCCCGGTTCTTT
CapZb_PCR_rev	CGTGGGCTTTACCAACACAG
CapZa1_Seq	GGTAGATACAGTCCTGCTATATGC
CapZa2_Seq	TTTACTGGTGTAAACTGGTCCAGG
CapZb_Seq	CAGGCTGCTCTATAAAGGGAAG
CPαY277Vβ fwd	CGACTGGAACAAGATACTCAGTGTCAAGATTGGCAAAGAAATGCAGAATGC
CPαY277Vβ rev	GCATTCTGCATTTCTTTGCCAATCTTGACACTGAGTATCTTGTTCCAGTCG
CPαW271Rβ fwd	GCAGCTTCCAGTTACCCGCACCAAAATCGACCGGAACAAGATACTCAG
CPαW271Rβ rv	CTGAGTATCTTGTTCCGGTCGATTTTGGTGCGGGTAACTGGAAGCTGC
CPαinsert fw	GAGAATCTTTATTTTCAGGGCGCCATGGCCGACTTTGAGGATCGGGTGTCG
CPαd8β rev	GGTGCTCGAGTGCGGCCGCTTATTACTTGTAACTGAGTATCTTGTTCCAG
CPαd9β rv	GGTGCTCGAGTGCGGCCGCTTATTAGTAACTGAGTATCTTGTTCCAGTCG

Supplementary Table 3. Primer sequences.

Supplementary Table 4

Key Resource Table				
**Reagent type (species) or resource**	**Designation**	**Source or reference**	**Identifiers**	**Additional Information**
Strain, strain background (*Escherichia coli*)	BL21 Rosetta	Novagen	Cat# 70954	Chemically competent cells
Strain, strain background (*Escherichia coli*)	BL21 Star pRARE	EMBL Protein Expression Facility		Chemically competent cells
Cell line (*mus musculus*)	B16F-1	ATCC	Cat# CRL-6323, RRID:CVCL_0158	CP quantifications by WB, CP KO and rescue experiments and EGFP-CP localization in vivo
Antibody	anti-CAPZA1 (mouse monoclonal)	BD Transduction Laboratories	612460	WB (1:5000)
Antibody	anti-CAPZB (mouse monoclonal)	Hybridoma bank USA[5]	3F2.3	WB (1:1000)
Antibody	anti-CAPZA1/2 (rabbit polyclonal)	Arigo Laboratories	Cat# ARG58328	WB (1:1000)
Antibody	Horseradish Peroxidase-conjugated anti-mouse IgG (goat polyclonal)	Dianova	115-035-062	WB (1:5000)
Antibody	anti-rabbit IgG (goat polyclonal)	Dianova	111-035-045	WB (1:5000)
Antibody	anti-ArpC5A (mouse monoclonal)	Home-made[5]	mAB323H3	IF (undiluted)
Antibody	anti-Mouse IgG (goat polyclonal)	Invitrogen	A-11032	IF (1:100)
Antibody	anti-HSC-70 (mouse monoclonal)	Santa Cruz	sc-7298	WB (1:10000)
Transfected construct	EGFP-CapZα1	This paper	Uniprot: P52907	Construct, can be obtained in the lab of P.

				Bieling, MPI Dortmund
Transfected construct	EG-CapZβ2	[6]	U10407.1	Construct can be obtained from Dorothy A Schafer University of Virginia, Charlottesville, VA, USA
Peptide, recombinant protein	Native bovine β, γ - actin	[7]		For cryo structure and biochemical experiments
Peptide, recombinant protein	Bovine Arp2/3 complex	This paper		For the generation of actin dendritic networks
Peptide, recombinant protein	Human myotrophin	This paper	Uniprot: P58546	Construct can be obtained in the lab of P. Bieling, MPI Dortmund
Peptide, recombinant protein	Mouse capping protein α1	[8]	Uniprot: 47753	Construct can be obtained in the lab of P. Bieling, MPI Dortmund
Peptide, recombinant protein	Mouse capping protein β2	[8]	Uniprot: 47757	Construct can be obtained in the lab of P. Bieling, MPI Dortmund
Peptide, recombinant protein	$UTRN_{261}$	[8]	Uniprot: P46939	Construct can be obtained in the lab of P. Bieling, MPI Dortmund
Peptide, recombinant protein	lifeact	[8]		Construct can be obtained in the lab of P. Bieling, MPI Dortmund
Peptide, recombinant protein	Human profilin 1	[7]	Uniprot: P07737	Construct can be obtained in the lab of P. Bieling, MPI Dortmund
Peptide, recombinant protein	Human WAVE1 WH_2 PVCA	[8]		Construct can be obtained in the lab of P. Bieling, MPI Dortmund
Peptide, recombinant protein	Human N-WASP	[8]		Construct can be obtained in the lab of P. Bieling, MPI Dortmund

Chemical compound, drug	Latrunculin B	Sigma Aldrich	Cat. #: L5288	For actin arrest
Chemical compound, drug	Phalloidin	Sigma Aldrich	Cat. #: P2141	For actin arrest
Chemical compound, drug	EZ-Link MaleimidePEG2-Biotin	Thermo Fisher	Cat. #: A39261	For NPF labeling
Chemical compound, drug	HO-PEG-NH2 and Biotin-CONH-PEG-OC3-H6-CONHS	Rapp Polymere	# 103000–20 and # 133000-25-35	For glass surface functionalization

Supplementary Table 4. Key resource table.

Supplementary References:

1. Scheres, S. H. W. RELION: implementation of a Bayesian approach to cryo-EM structure determination. *J Struct Biol* **180**, 519–530 (2012).
2. Tan, Y. Z. *et al.* Addressing preferred specimen orientation in single-particle cryo-EM through tilting. *Nat Methods* **14**, 793–796 (2017).
3. Beckers, M. & Sachse, C. Permutation testing of Fourier shell correlation for resolution estimation of cryo-EM maps. *J Struct Biol* **212**, 107579 (2020).
4. Urnavicius, L. *et al.* The structure of the dynactin complex and its interaction with dynein. *Science* **347**, 1441–1446 (2015).
5. Olazabal, I. M. *et al.* Rho-kinase and myosin-II control phagocytic cup formation during CR, but not FcgammaR, phagocytosis. *Curr Biol* **12**, 1413–1418 (2002).
6. Schafer, D. A. *et al.* Visualization and molecular analysis of actin assembly in living cells. *J Cell Biol* **143**, 1919–1930 (1998).
7. Funk, J. *et al.* Profilin and formin constitute a pacemaker system for robust actin filament growth. *Elife* **8**, (2019).
8. Bieling, P. *et al.* WH2 and proline-rich domains of WASP-family proteins collaborate to accelerate actin filament elongation. *EMBO J.* **37**, 102–121 (2018).
