# Supplementary material for: A barbed end interference mechanism reveals how capping protein promotes nucleation in branched actin networks
Source: Nat Commun. 2021 Sep 9;12:5329. doi: 10.1038/s41467-021-25682-5 (PMC8429771; doi:10.1038/s41467-021-25682-5)
Supplement: Supplementary file 3 — Reporting Summary [file 41467_2021_25682_MOESM3_ESM.pdf]

nature research

Corresponding author(s): Stefan Raunser and Peter Bieling

Last updated by author(s): Aug 4, 2021

Reporting Summary

Nature Research wishes to improve the reproducibility of the work that we publish. This form provides structure for consistency and transparency in reporting. For further information on Nature Research policies, see our Editorial Policies and the Editorial Policy Checklist.

Statistics

For all statistical analyses, confirm that the following items are present in the figure legend, table legend, main text, or Methods section.

n/a	Confirmed	
☐	☒	The exact sample size (*n*) for each experimental group/condition, given as a discrete number and unit of measurement
☐	☒	A statement on whether measurements were taken from distinct samples or whether the same sample was measured repeatedly
☐	☒	The statistical test(s) used AND whether they are one- or two-sided<br>*Only common tests should be described solely by name; describe more complex techniques in the Methods section.*
☒	☐	A description of all covariates tested
☐	☒	A description of any assumptions or corrections, such as tests of normality and adjustment for multiple comparisons
☐	☒	A full description of the statistical parameters including central tendency (e.g. means) or other basic estimates (e.g. regression coefficient) AND variation (e.g. standard deviation) or associated estimates of uncertainty (e.g. confidence intervals)
☐	☒	For null hypothesis testing, the test statistic (e.g. *F*, *t*, *r*) with confidence intervals, effect sizes, degrees of freedom and *P* value noted<br>*Give P values as exact values whenever suitable.*
☒	☐	For Bayesian analysis, information on the choice of priors and Markov chain Monte Carlo settings
☐	☒	For hierarchical and complex designs, identification of the appropriate level for tests and full reporting of outcomes
☒	☐	Estimates of effect sizes (e.g. Cohen's *d*, Pearson's *r*), indicating how they were calculated

Our web collection on statistics for biologists contains articles on many of the points above.

Software and code

Policy information about availability of computer code

Data collection

EPU Software was used for cryo data acquisition
Nikon Elements AR 4.50 and Micromanager 1.4 software (Edelstein et al., 2014) were used to acquire fluorescent images of in vitro assays.
MetaMorph7.8 software was used for image acquisition of cells.

Data analysis

Cryo image processing : was performed with SPHIRE 1.4 (Moriya et al., 2017). Filament ends were automatically detected with crYOLO 1.6.1 (Wagner et al., 2019). Images were denoised using the noise2noise implementation within cryolo (janni) and we further filtered the images using the ctfcorr.simple. filter within the e2proc2d.py program of EMAN2 2.9 (Tang et al., 2007). 2D classification was performed with ISAC 2.3.4 (Yang et al., 2012). Particles were exported to RELION 3.1 (Scheres, 2012) to perform Bayesian particle polishing (Zivanov et al., 2019). To estimate the directional FSC of the map we used the 3D-FSC package (Tan et al., 2017). Local resolution was estimated using the false-discovery rate FSC method implemented in SPOC (Beckers and Sachse, 2020). The final map was locally filtered using deepEMhancer Tensorflow version 1.14 (Sanchez-Garcia, 2020). We used MODELLER 10.1 (Eswar et al., 2008) to build a homology model of the complex between CP and the barbed end of human β-actin. To build an atomic model of the capped filament end, with fitted the initial homology model into the density using a combination of ISOLDE 1.2 (Croll, 2018) and Coot 0.9.5 (Emsley et al., 2010)

MetaMorph7.8 software was used for image analysis of cell experiments.

All microscopic and kinetic data from in vitro experiments were analyzed via kymograpgh analysis in Fiji 1.53C and plotted/ fitted in Origin9.0G as described in the methods section.

For manuscripts utilizing custom algorithms or software that are central to the research but not yet described in published literature, software must be made available to editors and reviewers. We strongly encourage code deposition in a community repository (e.g. GitHub). See the Nature Research guidelines for submitting code & software for further information.

Data

Policy information about availability of data

All manuscripts must include a data availability statement. This statement should provide the following information, where applicable:

- Accession codes, unique identifiers, or web links for publicly available datasets
- A list of figures that have associated raw data
- A description of any restrictions on data availability

All quantitative data presented in the manuscript are contained in the source data file, which is provided with this paper. Additional data are available from the corresponding authors upon request. A reporting summary for this Article is available as a Supplementary Information file. Our structure of capped barbed end is deposited in Protein Data Bank PDB, PDBID:7PDZ, EMDB-13343 [http://dx.doi.org/10.2210/pdb7pdz/pdb]. The previously published structure of the dynactin complex30 is available in the Protein Data Bank PDB, PDBID:5ADX [http://dx.doi.org/10.2210/pdb5adx/pdb]. The previously published structure of monomeric actin in complex with the WH2 domain of WASP51 is available in the Protein Data Bank PDB, PDBID: 2A3Z [http://dx.doi.org/10.2210/pdb2a3z/pdb].

Field-specific reporting

Please select the one below that is the best fit for your research. If you are not sure, read the appropriate sections before making your selection.

- [x] Life sciences
- [ ] Behavioural & social sciences
- [ ] Ecological, evolutionary & environmental sciences

For a reference copy of the document with all sections, see nature.com/documents/nr-reporting-summary-flat.pdf

Life sciences study design

All studies must disclose on these points even when the disclosure is negative.

Sample size	We did not use any statistical tests to predetermine sample size, but adhered to established practices in the field. The chosen number of filaments, network tails and cells was sufficiently large to yield mean values for each experimental replicate that were very similar.
Data exclusions	Data were not excluded from analysis
Replication	All results presented were replicated in at least three independent experiments. Replication attempts were successful and were consistent with result obtained by orthogonal methods where applicable.
Randomization	Actin networks, actin filaments and cells used for imaging were selected randomly from large data sets. All cells that passed quality control were analyzed equally with no sub-sampling and thus, there was no requirement for randomization.
Blinding	Investigators were not blinded during and data analysis or group allocation, since each experiment was performed by one researcher alone.<br><br>Blinding during group allocation was not possible to ensure that samples received the right experimental condition during the experiment.<br><br>Blinding was not performed during data analyis since data collection and analysis were performed by the same researcher.

Reporting for specific materials, systems and methods

We require information from authors about some types of materials, experimental systems and methods used in many studies. Here, indicate whether each material, system or method listed is relevant to your study. If you are not sure if a list item applies to your research, read the appropriate section before selecting a response.

Materials & experimental systems

n/a	Involved in the study	
☐	☒	Antibodies
☐	☒	Eukaryotic cell lines
☒	☐	Palaeontology and archaeology
☒	☐	Animals and other organisms
☒	☐	Human research participants
☒	☐	Clinical data
☒	☐	Dual use research of concern

Methods

n/a	Involved in the study	
☒	☐	ChIP-seq
☒	☐	Flow cytometry
☒	☐	MRI-based neuroimaging

Antibodies

Antibodies used	anti-CAPZB (mouse monoclonal, DSHB Cat# mAb 3F2.3) used 1:1000<br>anti-CAPZA1 (mouse monoclonal, BD Transduction Laboratories, Cat#612460) used 1:5000

	anti-CAPZA1/2 (rabbit polyclonal, Arigo Laboratories, Cat#ARG58328) used 1:1000<br>anti-ArpC5A (mouse monoclonal, Home-made (Olazabal et al., 2002), ID:mAB323H3) used undiluted<br>anti-mouse IgG (goat polyclonal, Dianova, Cat#115-035-062) used 1:5000<br>anti-rabbit IgG (goat polyclonal, Dianova, Cat#111-035-045) used 1:5000<br>Alexa Fluor 594 anti-mouse IgG (goat polyclonal, Invitrogen, Cat#A-11032) used 1:100<br>anti-HSC-70 (mouse monoclonal, Santa Cruz, Cat#sc-7298) used 1:10000
Validation	anti-CAPZB has been validated by Cooper J. A. succesfully by immunofluorescence staining (see PMID: 24319057/ 28899994)<br>anti-ArpC5A has been validated by Machesky L.M. successfully by by immunofluorescence staining (see PMID: 12194823)<br><br>All other antibodies were validated commercially. Certificates of analyis for the approved applications by the manufactures and references are available on the company websites as detailed below:<br><br>anti-CAPZA1: the antibody has been validated by BD Biosciences (https://www.bd.com/resource.aspx?IDX=12843) by immunoelectrophoresis, the antibody reacts with murine CAPZA1.<br><br>anti-CAPZA1/2: the antibody has been validated by Arigobio (https://www.arigobio.com/anti-CAPZA2-antibody-ARG58328.html) by immunoelectrophoresis using THP-1 cell lysate, the antibody reacts with both murine CAPZA1 and 2 isoforms.<br><br>anti-mouse IgG: the antibody has been validated by Dianova (https://www.dianova.com/en/shop/115-035-062-goat-igg-anti-mouse-igg-hl-hrpo-minx-huboho/) by immunoelectrophoresis and ELISA, the antibody reacts with the whole molecule mouse IgG<br><br>anti-rabbit IgG: the antibody has been validated by Dianova (https://www.dianova.com/en/shop/111-035-045-goat-igg-anti-rabbit-igg-hl-hrpo-minx-hu/) by immunoelectrophoresis and ELISA, the antibody reacts with whole molecule rabbit IgG<br><br>anti-HSC-70 : antibody has been validated by SantaCruz (https://www.scbt.com/p/hsc-70-antibody-b-6) by fluorescent western blot analysis of HSC 70 expression in K-562 (A), Hep G2 (B), MCF7 (C), C6 (D) and NIH/3T3 (E) whole cell lysates<br><br>Alexa Fluor 594 anti-mouse IgG: Antibody has been validated by Invitrogen (https://www.thermofisher.com/antibody/product/Goat-anti-Mouse-IgG-H-L-Highly-Cross-Adsorbed-Secondary-Antibody-Polyclonal/A-11032) by immunofluorescence on fixed and permeabilized HeLa cells.

Eukaryotic cell lines

Policy information about cell lines

Cell line source(s)	B16F-1 (ATCC, Cat# CRL-6323, RRID:CVCL_0158)
Authentication	authenticated via STR
Mycoplasma contamination	Cell lines were tested for mycoplasma contamination, no indication of contamination was observed
Commonly misidentified lines (See ICLAC register)	No commonly misidentified cell lines were used
